# Exploring the impact of key physicochemical properties of rice on taste quality and instant rice processing

**DOI:** 10.3389/fpls.2024.1481207

**Published:** 2024-11-07

**Authors:** Wen Yang, Xiaoling Li, Xiaohang Zheng, Mengyuan Wang, Wenxu Pan, Pin Liu, Zehua Zhang, Caixiong Gong, Ling Zheng, Hua Yuan, Ting Li, Weilan Chen, Peng Qin, Yuping Wang, Shigui Li, Bingtian Ma, Bin Tu

**Affiliations:** ^1^ Rice Research Institute of Sichuan Agricultural University, Chengdu, Sichuan, China; ^2^ State Key Laboratory of Crop Gene Exploration and Utilization in Southwest China, Chengdu, Sichuan, China; ^3^ Chongzhou Agriculture and Rural Bureau, Chengdu, Sichuan, China; ^4^ Hybrid Rice Research Center of Neijiang Academy of Agricultural, Neijiang, Sichuan, China

**Keywords:** CSSLs, amylose content, alkali spreading value, chalkiness, protein content, taste quality, instant rice

## Abstract

Taste quality is one of the most important indicators for assessing the quality of rice. However, there has been a lack of systematic studies investigating the impact factors of taste quality. In this study, chromosomal segment substitution lines (CSSLs) with notable differences in physicochemical properties were obtained by screening the CSSL population. A correlation analysis between the physicochemical properties and the taste qualities of rice revealed that amylose and protein content are significantly negatively correlated with the taste value of both freshly cooked and rehydrated instant rice. The alkali spreading value (ASV) had limited impact on the taste value of rice, but low-ASV rice is more resistant to cooking. Grain chalkiness played a critical role in maintaining the integrity of freshly cooked rice and instant rice grains after rehydration. In summary, our study provides crucial insights and guidance for rice breeding, with the goal of developing excellent quality and enhancing the processing of instant rice.

## Introduction

Rice stands as one of the world’s most vital cereal crops, serving as the primary source of essential nutrients and energy for over half of the global population, even in the face of dietary diversification trends (Buenafe et al., 2021; Fitzgerald et al., 2009). China is the world’s largest rice producer and consumer, accounting for approximately 40% of global rice production and consumption. While rice yields have seen significant advancements in recent decades, little progress has been achieved in rice quality improvement. Meanwhile, the demand for high-quality rice is increasing rapidly with rising incomes and improved living standards (Cheng et al., 2005). Rice quality encompasses various facets, including milling quality, grain appearance quality, eating and cooking quality (ECQ), and nutrition quality. Among these attributes, ECQ has emerged as the foundational characteristic, profoundly influencing consumer preferences and acceptance of rice (Li et al., 2023; Tu et al., 2022). ECQ paints a comprehensive picture that encompasses the appearance, aroma, taste, hardness, and viscosity of cooked rice grains. It encapsulates the sensory experience, involving sight, sound, smell, taste, and touch that rice imparts to individuals (Xu et al., 2013).

Considerable attention has been directed toward identifying the factors influencing the ECQ of rice (Hori et al., 2021; Xinkang et al., 2023; Yanjie et al., 2018). Traditionally, the gold standard for assessing ECQ has been descriptive sensory evaluation, which, in reality, is a time- and labor-consuming process reliant on trained sensory panelists (Tao et al., 2020). Recently, technological developments have provided alternative methods to evaluate rice ECQ including hardness viscosity analyzers capable of quantifying the physical characteristics of rice, yielding data on parameters such as hardness, viscosity, balance, and elasticity (Buenafe et al., 2021). The Rapid Visco Analyzer (RVA) is widely used to measure grain ECQ by evaluating viscosity indicators (Tong et al., 2014). The RVA spectra provide several characteristic parameters, including pasting temperature (PTm), peak viscosity (PV), trough viscosity (TV), final viscosity (FV), breakdown value (BDV), and setback value (SBV). Typically, higher BDV and lower SBV values are indicative of superior ECQ (Chen et al., 2021).

Rice quality is usually determined primarily by the measurement of physical and chemical properties. Foremost among these factors is the amylose content, with higher amylose content leading to a reduction in taste quality and increased firmness in the cooked rice (Tao et al., 2019). High-quality rice typically falls within the amylose content range of 13%–18% (Huang et al., 2020; Zeng et al., 2020). Amylose content is primarily regulated by the *Wx* gene, with multiple natural allele variants, including *Wx^lv^*, *Wx^a^*, *Wx^in^*, *Wx^b^*, and *wx* (ranked in descending order of amylose content). Researchers now generally believe that amylose content can be engineered through the exploration of *Wx* alleles (Zhang et al., 2019). In addition to amylose, protein content plays a dual role as a vital indicator of rice’s nutritional quality and an influence on taste quality. Multiple studies have demonstrated a significant negative correlation between protein content and rice taste quality (Yang et al., 2019; Zhang et al., 2021). Another critical factor is the *ALK* gene, which encodes soluble starch synthesis IIa (SSIIa), which is the major gene with at least three alleles, *ALK^G-TT^*, *ALK^A-GC^*, and *ALK^G-GC^*, linked to the diversity of ASV (Gao et al., 2011; Zheng et al., 2020). Meanwhile, chalkiness is an important cosmetic quality of rice which may also affect taste quality (Cheng et al., 2005). However, this approach has a significant drawback in explaining the overall ECQ, often deviating greatly from the actual measured ECQ.

There is currently a noticeable absence of systematic investigations into the impacts of these endosperm-stored substances on the ECQ of rice, particularly under similar genetic backgrounds. To address this knowledge gap, we turned to the CSSL population, which our lab previously constructed and which comprises chromosomal segments from 19 high-quality rice varieties (Hu et al., 2021). To delve into the effects of amylose content and ASV on ECQ, we identified CSSLs carrying three different alleles of *Wx* and *ALK^-GGC^* using polymorphic primers (Mikami et al., 2008; Zhang et al., 2019). We also screened CSSLs with high chalkiness, including high white-core rate (WCR) and white-belly rate (WBR), to estimate the effects of grain chalkiness on ECQ. Meanwhile, the effects of protein content on ECQ were investigated by different fertilization levels. In recognition of the increasing popularity of instant rice, we expanded our investigation to examine the effects of these important physicochemical properties on the processing and taste of instant rice. Our research serves as an important foundation, offering valuable insights and support for the breeding of rice varieties optimized for different cooking and processing methods.

## Materials and methods

### Plant materials and growth conditions

We screened a population of CSSLs (BC4F3) derived from the cross of R498 (as recipient parent) with 19 different donor parents using marker-assisted selection and chalkiness-related trait investigation. The parental line R498 and CSSLs were planted in Wenjiang in 2022. Nitrogen fertilizer treatment experiments were carried out in Wenjiang in 2021, consistent with a previous work (Li et al., 2023). Three varying levels of nitrogen fertilizer were applied: 0, 10, and 16 kg/666.7 m^2^. The nitrogen fertilizer was applied in two stages, with 70% applied as a base fertilizer and the remaining 30% at the tillering stage. For planting, the crops were organized into four rows, each consisting of 10 plants spaced at intervals of 26.7 × 16.7 cm. Field management adhered to established local agricultural practices.

### Measurement of agronomic traits

The agronomic traits were collected consistent with previous research (Hu et al., 2020; Tu et al., 2020). Five plants in the middle of each row were harvested and air-dried after maturation. The agronomic traits including plant height (PH), panicle length (PL), grain width (GW), grain length (GL), and 1,000-grain weight (KGW) were measured. GW, GL, and KGW were measured using SC-G seed counting and a grain weighing device (Wanshen Ltd., Hangzhou, China). Mean phenotypic values were compared using the Student’s *t*-test, and the data were analyzed using Microsoft Excel 2013.

### Amylose content and amylopectin chain-length distribution detection

For the determination of amylose content and amylopectin chain-length distribution, the milled rice was ground to rice flour with a grinder and sieved through a 0.5-mm mesh. The amylose content of the milled rice was measured using the standard iodine colorimetry method described in ISO 6647-2-2011 (Deng et al., 2018). The detailed operation is as follows: 0.01 g of sieved rice powder was placed into a 10-mL centrifuge tube. After 0.1 mL 95% ethanol was added, it was then gently shaken and mixed. Next, 0.9 mL 1 mol/L sodium hydroxide solution was added along the tube wall and mixed in. The centrifugal tube cover was tightly covered and placed at 30°C for 12 h for digestion. After digestion, 9 mL of distilled water was added to the centrifuge tube and mixed thoroughly. A 0.5-mL sample solution was added to a new 10-mL centrifuge tube, combined with 9 mL distilled water, 0.1 mL acetic acid, and 0.2 mL iodine, fully shaken and mixed, and then rested for 20 min. The blank solution was configured and used to adjust the wavelength of 620 nm to zero to measure the absorbance value of the colored sample solution.

Another portion of milled rice was used for the analysis of the amylopectin chain-length distribution, which was measured using a capillary electrophoresis fluorescent detection system (PA800 plus; Beckman-Coulter, CA, USA). From the original electropherogram scan, the peak area for each degree of polymerization (DP) was obtained, which is proportional to the number of chains. The DP was classified into four types following the method of Cameron et al.: A (6 ≤ DP ≤ 12), B1 (13 ≤ DP ≤ 24), B2 (25 ≤ DP ≤ 36), and B3+ (37 ≤ DP ≤ 60) (Cameron et al., 2007).

### Protein and lipid content detection

Protein content was measured from the total nitrogen content of head rice with a conversion index of 5.95 following the protocol of Deng et al. (2021). The crude fatty acid content in the rice grain power was measured by using the Soxhlet extraction method. In brief, 1 ± 0.05 g (m) of sieved rice flour in a filter paper bag was dried at 105°C for 3 h to a constant weight (m1). The crude fatty acids were extracted with petroleum ether for 90 min with ANKOMXT15i. Then, the filter paper bag containing the rice grain power was evaporated to dryness in an oven until a constant weight was obtained (m2). The fat content in the sample was calculated according to the following formula: (m1 - m2)/m × 100.

### Measurement of grain qualities

The dried seeds were processed into polished rice, and 100–200 polished rice grains from each plant were randomly selected to measure chalkiness. Images of head rice were captured, and chalkiness traits were measured with a Microtek ScanWizard EZ scanner and rice quality analyzer SC-E software (Hangzhou Wanshen Detection Technology Co., Ltd., Hangzhou, China; www.wseen.com). Viscosity was tested with Rapid Visco Analyzer (RVA) (Newport Scientific, Australia), and taste quality was estimated using Rice Taste Meter (KETT, Japan) following the method of Tu et al. (2022). Excel 2013 and SPSS v19.0 were used for data collation and analysis.

### Taste quality detection of instant rice

To prepare the rice for analysis, 30 g of polished rice grains was washed three times and soaked for 10 min. Then, 1.4 times the resulting volume of distilled water was added, cooked for 20 min, and then stewed for 10 min. The cooked rice was then cooled to ambient room temperature and dried using hot air at 80°C for 3 h. The instant rice was prepared by adding 1.2 times the volume of boiled water and incubated for 10 min. The taste quality of the freshly cooked and rehydrated instant rice was detected as described in “Measurement of grain qualities”.

## Results

### Isolation of the CSSLs, investigation of agronomic traits, and genomic sequencing analysis

**Figure 1:**
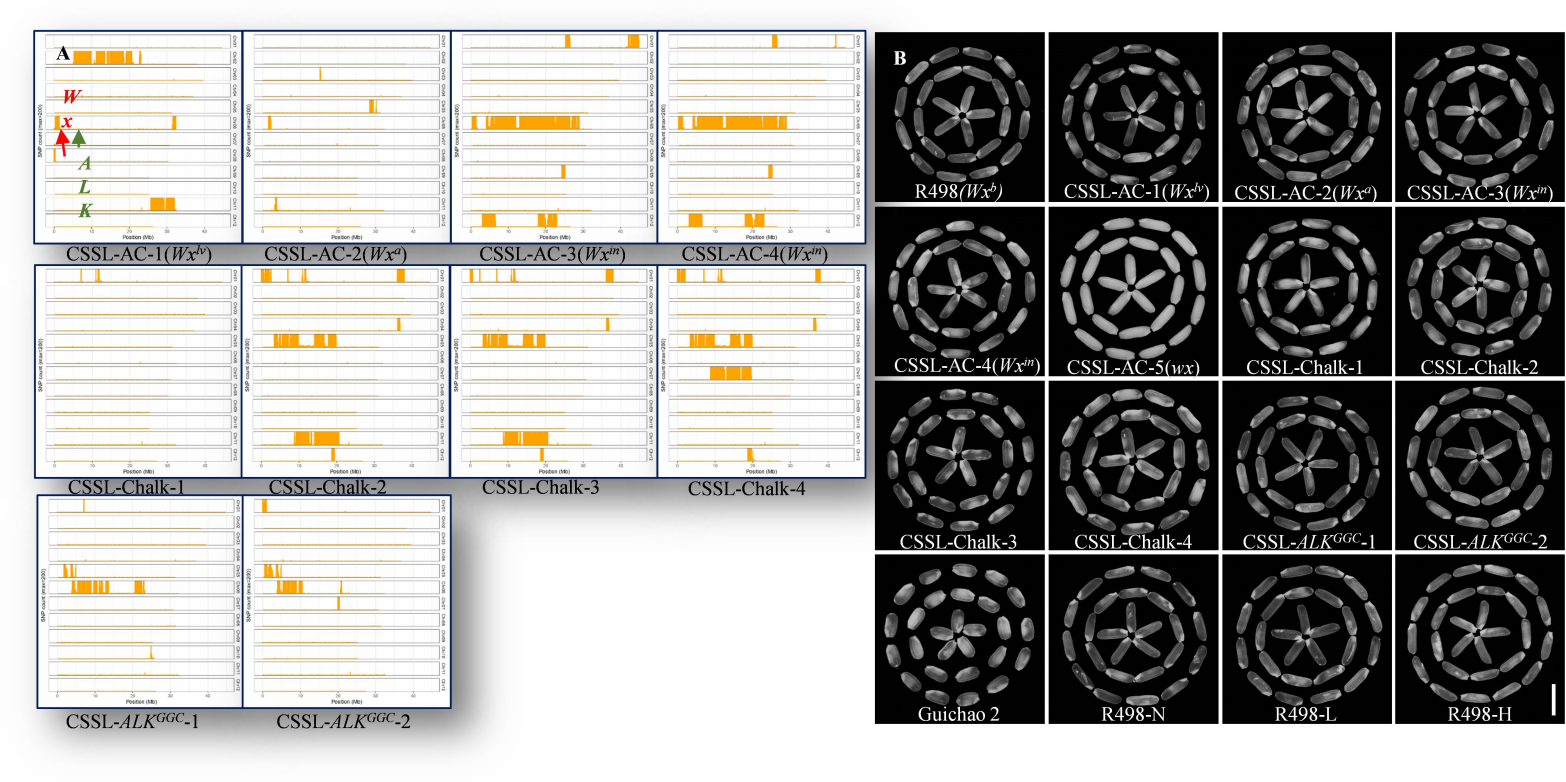
Diagram of genome fragment substitution based on genomic sequencing and polished grains of chromosomal segment substitution lines (CSSLs) and R498. **(A)** Diagram of genome fragment substitution, with R498 as the reference genome. **(B)** Polished grains of isolated CSSLs. Bar = 10 mm. R498-N, R498-L, and R498-H are the R498 plants with normal, low, and high nitrogen application, respectively.

In order to explore the effects of amylose content, ASV, and grain chalkiness on the ECQ of rice, we screened a CSSL population previously constructed in our laboratory to identify CSSLs carrying different alleles of *Wx* and *ALK* genes and CSSLs with high grain chalkiness. The process of selecting segment substitution lines involved careful screening based on specific criteria. Agronomic traits such as plant height, growth rate, yield potential, disease resistance, and stress tolerance were assessed in the field. This allowed for the identification of lines that exhibited desirable traits for further analysis. In total, we obtained five CSSLs containing *Wx^lv^*, *Wx^a^*, *Wx^in^*, and *wx* alleles and two CSSLs carrying *ALK^G-GC^* allele with low ASV. In addition, we identified four CSSLs with high chalkiness, including one CSSL with high WCR and three CSSLs with high WBR, which share identical *Wx* and *ALK* genotype as the receptor parent, R498 (**Figures 1A, B**; **Supplementary Table S1**).

We investigated the main agronomic traits including plant height (PH), panicle length (PL), tiller number (TN), seed setting rate (SSR), 1,000 grain weight (KGW), grain length (GL), and grain width (GW) of R498 and each CSSL. Our data reveal multiple variations of main agronomic traits between the CSSLs and R498, among which the differences in grain-shape-related traits, including KGW, GL, and GW, were the most obvious, which may be caused by the imported chromosome fragments containing the major genes of these agronomic traits (**Supplementary Table S1**; **Figure 1A**). Generally, the plant morphology of each CSSL was similar to that of 498 (**Supplementary Figure S1**), consistent with the results that the genetic backgrounds of all the CSSLs are relatively clear. Gui Chao 2 is a high-yield rice variety with wide adaptability, but its appearance quality and ECQ are poor and used as a negative control in this study (**Figure 1B**; **Supplementary Table S2**).

Genomic sequencing analysis was conducted to unravel the genetic makeup of the CSSLs. High-throughput sequencing technologies were employed to obtain comprehensive genomic data, with R498 as the reference genome. The fragment substitutions of CSSLs on each chromosome are shown in **Figure 1A**, in which each genome is divided into 0.01-Mb fragments. The position information of the mutation site and the number of SNPs that differ between each fragment and R498 were calculated. The genome infiltration of each CSSL was assessed, and it revealed that the backgrounds of all the CSSLs are relatively pure (**Supplementary Figure S1**). Both *Wx* and *ALK* genes located on chromosome 6 can be excluded from the four CSSLs with high chalkiness (**Figure 1A**).

Earlier studies have revealed that *ALK* is the major gene controlling ASV variation between *indica* and *japonica* rice. ALK encodes starch synthase IIa (SSIIa) and plays a key role in the synthesis of long B1 chains by extending the short A and B1 chains of amylopectin in the endosperm (Gao et al., 2003, 2011; Nakamura et al., 2005; Yu et al., 2011). The *indica* allele *ALK^i^* (G-GC) controlled lower ASV, and inactive *japonica* alleles *ALK^j^* (A-GC or G-TT) controlled high ASV (Fan et al., 2017; Gao et al., 2011; Wang et al., 2015).

**Figure 2:**
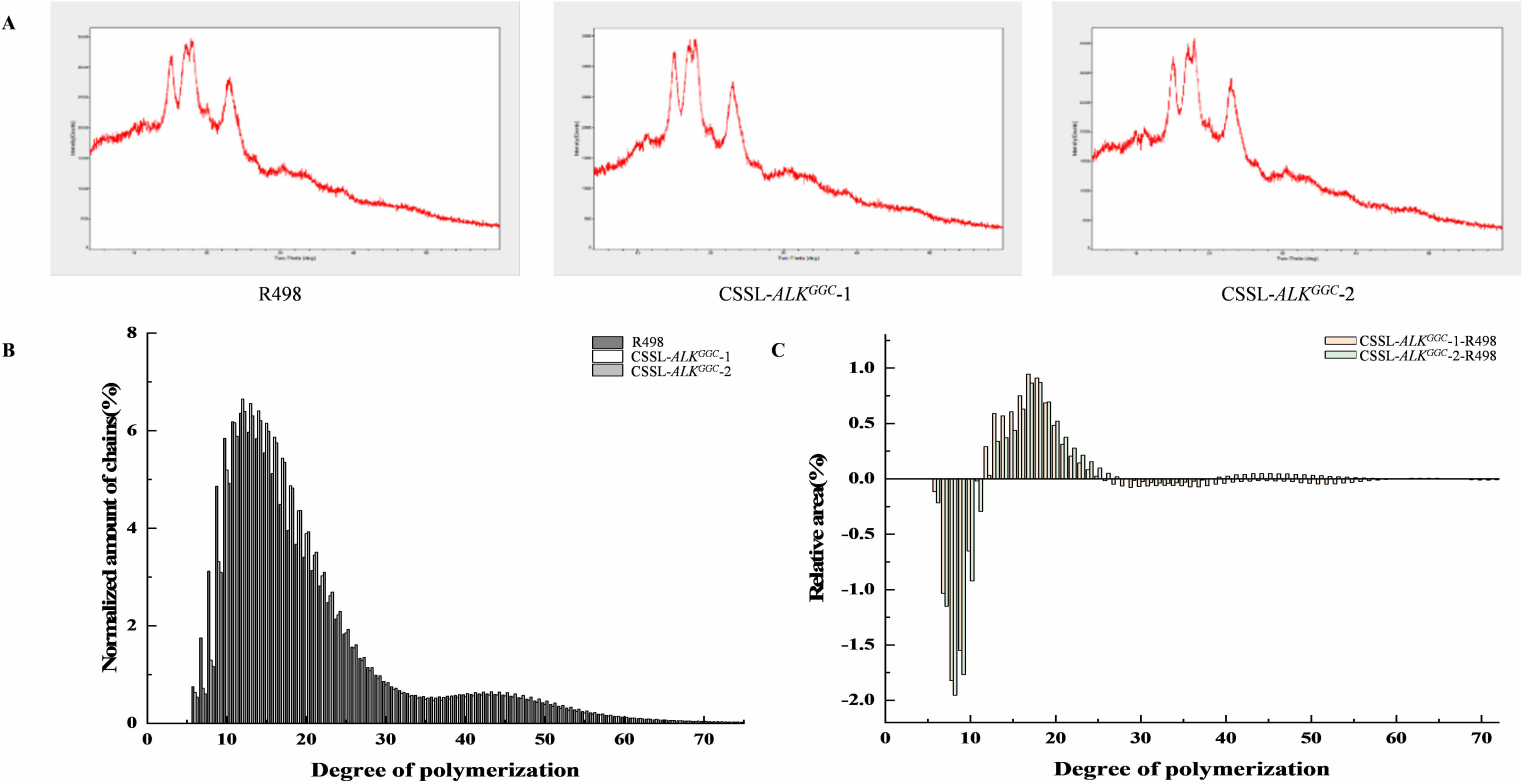
Property and chain-length detection of amylopectin in two chromosomal segment substitution lines (CSSLs) with low ASV. **(A)** XRD patterns of rice starch from R498 and two CSSLs with *ALK^GGC^* allele. **(B, C)** The normalized amount **(B)** and relative amount **(C)** of amylopectin chain-length distribution of R498 and two CSSLs with *ALK^GGC^* allele.

To investigate the impact of introducing the *ALK^GGC^* allele into R498 (*ALK^GTT^*) on the physicochemical properties of amylopectin, the XRD pattern and amylopectin branching chain-length distribution of these two CSSLs were detected in collaboration with Sanshubio (http://www.sanshubio.com/). Both of the CSSLs displayed typical A-type diffraction patterns (**Figure 2A**). Compared to R498, these two substitution lines showed a decrease in the number of A chains and B2 chains, while the number of B1 chains increased (**Figures 2B, C**). This could be attributed to the promotion of A chains into B1 chains by SSIIa, resulting in an increase in rice gelatinization temperature (Nakamura et al., 2005).

### Accuracy test of rice taste meter

To test the accuracy of the rice taste meter and hardness viscosity analyzer, we selected five rice varieties for testing: Yixiangyou 2115 (YX2115), Huihe 5A/R584 (HY584), Quanxiang 3A/R612 (QXY612), Fyou 498 (FY498), and Gui Chao 2. These rice varieties were chosen to represent a sequential decrease in taste quality as determined by sensory evaluation results. The ECQ of these five rice varieties was detected by the rice taste meter and hardness viscosity analyzer, and the comprehensive taste scores were revealed to be consistent with the results of the sensory evaluation (**Supplementary Table S2**). The rice varieties with high comprehensive scores had better color, higher appearance, viscosity, balance, and taste scores and lower hardness. A correlation analysis of the measured hardness, viscosity, balance, elasticity and comprehensive taste score was carried out, and the results showed that the comprehensive taste core was negatively correlated with hardness and positively correlated with viscosity and balance (**Supplementary Table S3**). In short, the rice taste meter and hardness viscosity analyzer can accurately determine the ECQ of rice.

### Determination and correlation analysis of the physicochemical characteristics and ECQs of each isolated CSSL

Our analysis of appearance quality and the main nutrient content revealed significant findings. Specifically, CSSLs containing *Wx^lv^*, *Wx^a^*, and *Wx^in^* exhibited a notably higher amylose content than R498, and their chalky-grain percentage (CGP) and chalky-grain grade (CGG) were significantly higher than those of R498 (**Table 1**), consistent with recent findings that the *Wx* genotype may be associated with grain chalkiness formation and rice varieties with *Wx^a^* and *Wx^in^* alleles produced more chalky grains (Li et al., 2023; Xia et al., 2022). CSSL-AC-5 carrying the *wx* allele is a glutinous rice with a milky-white grain. The CSSLs with high chalkiness showed a decrease in amylose content overall, while the protein and lipid contents tended to increase (**Table 1**), perhaps due to the lack of tight filling of nutrient materials (Lin et al., 2014; Liu et al., 2011). In contrast, CSSLs with lower ASV exhibited lower CGP than R498 (**Table 1**).

**Table 1:** Determination of grain appearance quality, nutrient content, and eating and cooking-related quality of each chromosomal segment substitution line.

Materials	CGP (%)	CGG (%)	PC (%)	FC (%)	AC (%)	Comprehensive	Appearance	Taste	Hardness (kgf)	Viscosity (kgf)	Balance	Elasticity
R498(*Wx^b^*)	49.87 ± 0.11	18.31 ± 2.54	5.17 ± 0.29	0.32 ± 0.05	17.69 ± 0.31	88.00 ± 1.00	8.53 ± 0.21	8.00 ± 0.26	2.23 ± 0.43	0.38 ± 0.05	0.17 ± 0.02	0.88 ± 0.01
CSSL-AC-1(*Wx^lv^*)	88.50 ± 0.02**	31.49 ± 2.21**	5.85 ± 0.29*	0.44 ± 0.00	28.22 ± 0.32**	74.67 ± 0.58**	7.50 ± 0.10**	6.50 ± 0.10**	4.39 ± 0.08**	0.04 ± 0.01**	0.01 ± 0.00**	0.90 ± 0.01*
CSSL-AC-2(*Wx^a^*)	82.57 ± 0.02**	58.78 ± 2.32**	5.87 ± 0.13*	0.35 ± 0.16	24.18 ± 0.58**	82.25 ± 3.10*	7.90 ± 0.36*	7.05 ± 0.34*	3.21 ± 2.05	0.10 ± 0.02**	0.03 ± 0.01**	0.90 ± 0.01*
CSSL-AC-3(*Wx^in^*)	91.53 ± 0.02**	45.44 ± 0.32**	6.75 ± 0.06**	0.52 ± 0.16	23.11 ± 0.20**	79.00 ± 1.00**	7.50 ± 0.17**	6.93 ± 0.23**	3.32 ± 0.32*	0.11 ± 0.03**	0.03 ± 0.02**	0.92 ± 0.04
CSSL-AC-4(*Wx^in^*)	93.87 ± 0.02**	62.22 ± 1.83**	5.41 ± 0.07	0.56 ± 0.19	22.40 ± 0.42**	81.67 ± 1.53**	7.87 ± 0.23*	7.27 ± 0.23**	3.63 ± 0.26**	0.13 ± 0.02**	0.04 ± 0.01**	0.92 ± 0.07
CSSL-AC-5(*wx*)	100.00 ± 0.00**	100.00 ± 0.00**	6.00 ± 0.20*	0.56 ± 0.19	0.76 ± 0.41**	88.00 ± 0.00	8.37 ± 0.15**	8.27 ± 0.06**	1.04 ± 0.11**	0.38 ± 0.08	0.36 ± 0.04**	0.88 ± 0.01
CSSL-Chalk-1	100.00 ± 0.00**	59.89 ± 0.92**	6.07 ± 0.10**	1.43 ± 0.15**	12.61 ± 0.10**	87.67 ± 0.58	8.67 ± 0.06	8.10 ± 0.17	1.57 ± 0.18	0.54 ± 0.07*	0.35 ± 0.02**	0.88 ± 0.04
CSSL-Chalk-2	93.57 ± 0.08**	29.64 ± 1.76**	6.27 ± 0.02**	0.94 ± 0.02**	14.97 ± 0.40**	88.67 ± 0.58	8.73 ± 0.06	8.17 ± 0.13	1.94 ± 0.23	0.49 ± 0.05	0.25 ± 0.01**	0.88 ± 0.01
CSSL-Chalk-3	98.10 ± 0.03**	47.59 ± 1.95**	6.11 ± 0.05**	0.75 ± 0.05*	15.26 ± 0.38**	88.67 ± 1.15	8.90 ± 0.00*	8.60 ± 0.69	2.25 ± 0.18	0.62 ± 0.08*	0.27 ± 0.04*	0.86 ± 0.02
CSSL-Chalk-4	94.63 ± 0.02**	60.61 ± 2.67**	5.67 ± 0.11*	0.58 ± 0.04*	14.11 ± 0.40**	90.00 ± 1.00	8.93 ± 0.15	8.53 ± 0.25	2.07 ± 0.12	0.38 ± 0.06	0.18 ± 0.02	0.85 ± 0.03
CSSL-*ALK^GGC^*-1	40.72 ± 0.96**	16.99 ± 0.49**	4.44 ± 0.61	0.40 ± 0.24	15.65 ± 0.41**	90.00 ± 1.00	8.67 ± 0.21	8.47 ± 0.31	2.24 ± 0.54	0.58 ± 0.17	0.26 ± 0.03*	0.87 ± 0.04
CSSL-*ALK^GGC^*-2	42.76 ± 1.90**	24.40 ± 0.89**	4.44 ± 0.11	0.39 ± 0.02	16.08 ± 1.03*	92.33 ± 0.58**	9.33 ± 0.12**	9.40 ± 0.17**	1.73 ± 0.32	0.45 ± 0.09	0.26 ± 0.02**	0.88 ± 0.02
Guichao 2	100.00 ± 0.00**	61.69 ± 1.85**	7.43 ± 0.27**	0.24 ± 0.03	25.41 ± 0.34**	53.33 ± 1.53**	5.03 ± 0.21	4.47 ± 0.15**	9.05 ± 0.43**	0.12 ± 0.03**	0.01 ± 0.01**	0.77 ± 0.01**
R498-N	41.28 ± 0.18	16.81 ± 0.45**	4.27 ± 0.15**	0.48 ± 0.03	16.08 ± 0.47**	91.67 ± 0.58**	9.00 ± 0.10*	8.93 ± 0.15**	2.44 ± 0.36	0.64 ± 0.09*	0.26 ± 0.04*	0.84 ± 0.01**
R498-L	46.87 ± 2.04**	16.96 ± 0.63**	4.30 ± 0.13**	0.30 ± 0.20	15.54 ± 0.00**	89.67 ± 0.58	8.80 ± 0.10	8.50 ± 0.20	2.69 ± 0.24	0.54 ± 0.08*	0.22 ± 0.01*	0.88 ± 0.06
R498-H	35.83 ± 3.01**	24.15 ± 0.95**	6.26 ± 0.20**	0.49 ± 0.24	18.25 ± 0.40	86.67 ± 0.58	8.57 ± 0.06	7.97 ± 0.12	2.41 ± 0.34	0.47 ± 0.12	0.19 ± 0.02	0.89 ± 0.05

* and ** denote significant differences at the 0.05 and 0.01 levels, respectively.

**Figure 3:**
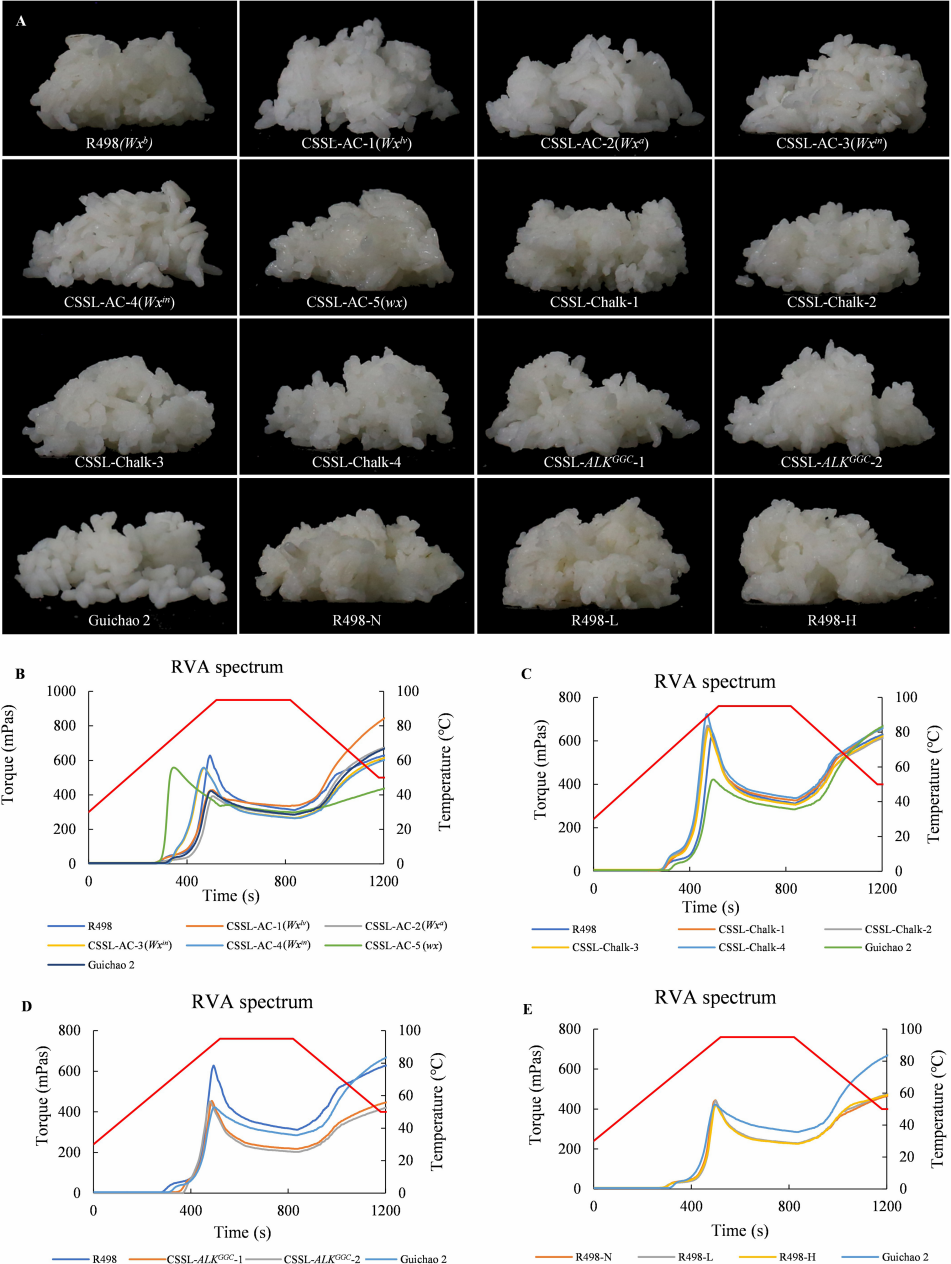
Effects of the main physicochemical properties on the appearance of cooked rice and starch gelatinization characteristics. **(A)** Appearance of cooked rice of each chromosomal segment substitution line (CSSL). **(B–E)** Rapid Visco Analyzer spectrum of CSSLs with different AC **(B)**, chalkiness **(C)**, alkali spreading value **(D)**, and protein content **(E)**.

Throughout the rice preparation process, the cooked rice grains from the four CSSLs with high chalkiness, two CSSLs with lower ASV, and R498 under varying fertilization conditions all exhibited excellent grain integrity and color (**Figure 3A**). Rice with low to medium levels of amylose, such as CSSL-AC-5 (*wx*), a glutinous rice, and R498 (*Wx^b^*), attained the highest taste quality scores (**Table 1**). Conversely, CSSLs with higher amylose levels, such as CSSL-AC-1 (*Wx^lv^*), CSSL-AC-2 (*Wx^a^*), CSSL-AC-3 (*Wx^in^*), and CSSL-AC-4 (*Wx^in^*), exhibited lower scores across categories of appearance, taste, and comprehensive taste quality (**Table 1**). Furthermore, we observed that cooked rice grains from CSSLs with elevated amylose content displayed greater dispersion, akin to Gui Chao 2, a rice variety known for its lower taste quality (**Figure 3A**). In conclusion, the amylose content of rice has a substantial impact on both its appearance and eating and cooking quality (ECQ). The comprehension and manipulation of amylose content can yield valuable insights to enhance the overall quality and consumer acceptance of rice varieties (Tian et al., 2009; Xu et al., 2021; Zhang et al., 2021).

This investigation revealed a significant correlation between nitrogen application and the protein content in R498 grains. As the nitrogen levels increased, protein content also rose markedly. However, this increase in protein content was accompanied by a gradual reduction in viscosity and balance of appearance, resulting in diminished taste quality and lower comprehensive taste scores. Under the condition of no nitrogen fertilizer application, the comprehensive taste scores, appearance, and taste scores all showed improvements, along with enhanced viscosity and balance. Importantly, there was no significant change in hardness, resulting in the best taste quality. Conversely, under high nitrogen levels, taste quality was at its lowest, with a decrease in viscosity and poorer balance (**Table 1**). This outcome aligns with the observation that as protein content increases, the taste value of rice decreases. It is worth noting that protein content is a vital indicator to evaluate the nutritional quality of rice, in line with prior research findings (Zhang et al., 2020).

Interestingly, the CSSLs with high chalkiness had high comprehensive taste scores, but their cooked rice grains were more fragile, particularly those from CSSL-Chalk-1, which had a high WCR (**Figure 3A**; **Table 1**). Therefore, chalkiness is one of the factors that determine the appearance quality of rice. The two low-ASV CSSLs had higher appearance and taste scores, and there was an upward trend in the comprehensive taste scores (**Table 1**). Thus, within the context of R498, ASV appears to exert minimal influence on taste and predominantly affects the gelatinization temperature of rice.

The correlation analysis revealed a significant negative correlation between amylose content, protein content, and taste quality. Amylose content has the strongest correlation with taste quality and the greatest impact on taste, followed by protein content (**Supplementary Table S4**). On the other hand, chalkiness shows a weak correlation with taste qualities.

### Effect of rice physicochemical characteristics on starch gelatinization characteristics

The RVA measurement results of materials with different amylose contents are shown in **Figure 3B**. CSSL-AC-5 with the *wx* allele exhibits low gelatinization temperature and peak viscosity (PV), and its breakdown value (BDV) was greater than its setback value (SBV), resulting in good taste (**Figure 3B**). The RVA curves of CSSL-AC-1 (*Wx^lv^*) and CSSL-AC-2 (*Wx^a^*) with amylose content higher than 25% are similar to that of the negative control Gui Chao 2 (**Figure 3B**), while CSSL-AC-3 and CSSL-AC-4 with the *Wx^in^* alleles had amylose content ranging from 18% to 22%. They show high final viscosity (FV) with significantly greater consistency values (CSV) than BDV, indicating a high retrogradation tendency (**Figure 3B**).

The CSSLs with high chalkiness have higher PV compared to the negative control Gui Chao 2 and the control R498. Their FVs are lower than the negative control Gui Chao 2. The gelatinization temperature is lower than that of Gui Chao 2, with negative SBVs and BDVs higher than CSVs, resulting in better taste. Thus, chalkiness appears to have a relatively weak impact on taste (**Figure 3C**; **Supplementary Table S4**).

The CSSLs with lower ASV exhibit lower PV and FV values, along with positive SBVs that do not show significant variation. This pattern indicates a reduced tendency for retrogradation in the rice (**Figure 3D**). The RVA profile of CSSL-*ALK* ^GGC^-2 shows a higher gelatinization temperature compared to CSSL-*ALK* ^GGC^-1, suggesting that the starch granules in CSSL-*ALK* ^GGC^-2 are more difficult to gelatinize. Compared to CSSL-*ALK* ^GGC^-2, CSSL-*ALK* ^GGC^-1 has no significant changes in PV and trough viscosity, but has higher FV, resulting in a larger CSV and SBV and relatively poorer taste (**Figure 3D**).

Among the nitrogen fertilizer treatments, the RVA curves of the three different nitrogen application levels are similar. In comparison to the negative control Gui Chao 2, they generally exhibit lower TV and FV, with the CSV being smaller than the BDV, indicating a relatively poorer taste quality (**Figure 3E**). Of the three nitrogen application levels, the treatment without nitrogen fertilizer stood out with the lowest SBV and the best taste (**Figure 3E**). In the high nitrogen treatment, the PV is low, and the SBV is the highest, resulting in the worst taste quality (**Figure 3E**).

### Predictive criteria for indica rice taste quality

As amylose content increases, the SBV and retrogradation tendency of starch after gelatinization also rise, leading to a decline in taste quality. On the other hand, ASV primarily influences the gelatinization temperature of starch, with higher ASV resulting in lower gelatinization temperature. Different nitrogen application levels primarily affect the PV during starch gelatinization. Rice subjected to high nitrogen levels exhibits the lowest PV, smaller BDV, and larger SBV and CSV, resulting in variations in taste quality. Based on these findings, we propose an ECQ evaluation criterion for indica rice, which includes a direct amylose content of around 13%–17%, a low degree of chalkiness, and low protein content.

To validate this ECQ evaluation criterion, we conducted laboratory tests on 10 selected rice varieties to assess the impact of various physicochemical characteristics on ECQs, including amylose content, chalkiness, and ASV, as measured by the China National Rice Research Institute (**Supplementary Table S5**). The results showed that the hybrid rice varieties Jincheng 3A/Shuhui 91 and Jincheng 2A/Shuhui 91, which had higher amylose content compared to other materials, had lower viscosity and poorer balance, resulting in lower taste scores. The hybrid rice varieties Quanxiang 3A/Shuhui 882, Quanxiang 1A/Shuhui 882, and Quanxiang 3A/Shuhui 569, which had higher protein content, exhibited consistent ASV, chalkiness ratios, and amylose content, leading to lower taste scores and lower appearance and texture scores with higher hardness. The hybrid rice variety S28-13/Yuhe had the highest taste score, with an amylose content of around 16% and lower protein content and ASV. This further confirmed that increasing amylose and protein content decreases the taste quality of rice, while the impact of ASV on taste quality was limited.

### Effects of different physicochemical characteristics of rice on instant rice processing

Firstly, we conducted a comparison between hot air drying and freeze-drying to assess their impact on the rehydration texture of instant rice. It was observed that grains dried by hot air exhibited a slightly yellowish appearance with cracks on the surface, while the freeze-dried grains were paler and plump, although the freeze-drying process took a longer time. Consequently, we selected hot air drying for subsequent experiments.

Secondly, we performed sensory evaluations on three different rehydration ratios (1:1, 1:1.2, and 1:1.4) for instant rice. The results indicated that using a 1:1 water-to-rice ratio did not lead to complete water absorption, resulting in an uneven texture. Conversely, the rice with a 1:1.4 water-to-rice ratio became excessively soft due to an excess of water. In contrast, the rice with a 1:1.2 water-to-rice ratio exhibited a shiny appearance and effectively absorbed the added water. As a result, we recommend a rehydration ratio of 1:1.2 for the preparation of instant rice.

**Figure 4:**
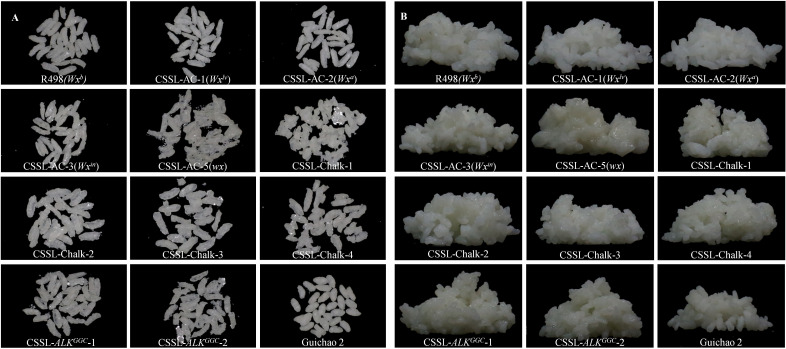
Appearance comparison of instant rice made from different chromosomal segment substitution lines (CSSLs) after drying and rehydration. **(A)** Dried instant rice grains of different CSSLs. **(B)** Rehydrated instant rice of different CSSLs.

The assessment of the grain integrity after drying and taste quality of rehydrated instant rice for different CSSLs revealed that materials with a higher amylose content, such as those carrying *Wx^lv^* and *Wx^a^* alleles, had better grain integrity but poorer taste quality compared to directly steamed rice (**Table 2**). On the other hand, R498 and glutinous rice materials with medium to low amylose content exhibited relatively poorer grain integrity after drying, but there was no significant decrease in palatability after rehydration (**Figure 4A**; **Table 2**). The impact of chalkiness on the taste quality of rehydrated instant rice is relatively weak (**Table 2**), but different types of chalkiness have different effects on the grain integrity after drying. A CSSL with high WCR is more likely to result in incomplete and fragile rice grains (**Figure 4A**). Therefore, it is advisable to select varieties with lower chalkiness, especially lower WCR, for instant rice production.

**Table 2:** Eating and cooking quality of freshly cooked rice and rehydrated instant rice of each chromosomal segment substitution line.

Materials	Comprehensive		Appearance		Taste		Hardness		Viscosity		Balance			Elastic
	Freshly cooked	Rehydration	Freshly cooked	Rehydration	Freshly cooked	Rehydration	Freshly cooked	Rehydration	Freshly cooked	Rehydration	Freshly cooked	Rehydration	Freshly cooked	Rehydration
R498(*Wx^b^*)	91.00 ± 0.00	88.33 ± 1.53**	9.10 ± 0.14	8.57 ± 0.31**	8.65 ± 0.07	7.77 ± 0.47**	2.17 ± 0.13	1.83 ± 0.19**	0.54 ± 0.00	0.41 ± 0.01**	0.25 ± 0.01	0.22 ± 0.02	0.90 ± 0.01	0.88 ± 0.05
CSSL-AC-1(*Wx^lv^*)	77.33 ± 1.15	67.00 ± 1.73**	7.73 ± 0.12	6.43 ± 0.12**	6.63 ± 0.12	5.40 ± 0.17**	5.42 ± 0.37	4.68 ± 0.03**	0.04 ± 0.02	0.01 ± 0.01**	0.01 ± 0.01	0.00 ± 0.00	0.88 ± 0.00	0.86 ± 0.02
CSSL-AC-2(*Wx^a^*)	75.67 ± 2.52	65.67 ± 4.04**	7.63 ± 0.25	6.33 ± 0.51**	6.53 ± 0.25	5.23 ± 0.51**	5.69 ± 0.11	4.94 ± 0.26**	0.04 ± 0.01	0.01 ± 0.01**	0.01 ± 0.00	0.00 ± 0.00	0.87 ± 0.02	0.84 ± 0.02
CSSL-AC-3(*Wx^in^*)	82.00 ± 1.73	79.33 ± 1.15**	7.97 ± 0.12	7.77 ± 0.15*	7.20 ± 0.17	7.03 ± 0.15*	3.36 ± 0.11	2.69 ± 0.33*	0.36 ± 0.06	0.30 ± 0.07**	0.11 ± 0.03	0.11 ± 0.01	0.87 ± 0.01	0.86 ± 0.03
CSSL-AC-4(*Wx^in^*)	82.67 ± 1.15	80.33 ± 1.53**	7.87 ± 0.12	7.50 ± 0.17**	7.10 ± 0.10	6.90 ± 0.17*	3.50 ± 0.27	3.14 ± 0.10**	0.42 ± 0.14	0.29 ± 0.08**	0.12 ± 0.04	0.09 ± 0.01	0.88 ± 0.03	0.84 ± 0.01
CSSL-AC-5(*wx*)	89.67 ± 0.58	89.67 ± 0.58	8.80 ± 0.00	8.67 ± 0.12	8.17 ± 0.06	8.10 ± 0.10	1.15 ± 0.03	0.70 ± 0.08**	0.42 ± 0.05	0.22 ± 0.05**	0.37 ± 0.05	0.32 ± 0.03	0.89 ± 0.01	0.91 ± 0.02
CSSL-Chalk-1	86.33 ± 0.58	85.67 ± 0.58*	8.33 ± 0.06	8.10 ± 0.17	7.47 ± 0.06	7.40 ± 0.17	2.07 ± 0.16	1.38 ± 0.11*	0.64 ± 0.09	0.43 ± 0.06**	0.31 ± 0.03	0.31 ± 0.06	0.86 ± 0.02	0.86 ± 0.02
CSSL-Chalk-2	89.33 ± 0.58	88.67 ± 0.58	9.00 ± 0.00	8.77 ± 0.15*	8.33 ± 0.06	8.03 ± 0.29**	2.28 ± 0.21	1.77 ± 0.04**	0.64 ± 0.09	0.45 ± 0.04**	0.28 ± 0.03	0.25 ± 0.04	0.88 ± 0.02	0.88 ± 0.03
CSSL-Chalk-3	88.33 ± 0.58	89.33 ± 0.58	8.80 ± 0.00	8.83 ± 0.15	7.93 ± 0.06	8.10 ± 0.26	2.28 ± 0.15	2.05 ± 0.30**	0.54 ± 0.08	0.48 ± 0.03**	0.24 ± 0.03	0.23 ± 0.02	0.90 ± 0.02	0.86 ± 0.01
CSSL-Chalk-4	90.00 ± 0.00	89.67 ± 0.58	9.03 ± 0.06	8.97 ± 0.15	8.47 ± 0.12	8.40 ± 0.17	2.34 ± 0.21	1.71 ± 0.09**	0.58 ± 0.13	0.52 ± 0.03*	0.25 ± 0.03	0.30 ± 0.04*	0.89 ± 0.01	0.90 ± 0.01
CSSL*-ALK^GGC^*-1	87.67 ± 0.58	89.00 ± 0.00*	8.73 ± 0.12	8.70 ± 0.00	8.07 ± 0.15	8.23 ± 0.06*	2.14 ± 0.38	1.56 ± 0.06**	0.53 ± 0.11	0.49 ± 0.09**	0.25 ± 0.04	0.32 ± 0.02**	0.88 ± 0.01	0.89 ± 0.02
CSSL-*ALK^GGC^*-2	89.67 ± 0.58	91.00 ± 0.00*	8.93 ± 0.12	9.03 ± 0.06	8.37 ± 0.15	8.70 ± 0.10**	2.17 ± 0.23	1.41 ± 0.09**	0.49 ± 0.08	0.43 ± 0.06	0.23 ± 0.02	0.31 ± 0.04*	0.88 ± 0.01	0.88 ± 0.01
Guichao 2	66.67 ± 2.52	62.67 ± 2.89**	6.37 ± 0.32	5.90 ± 0.36**	5.43 ± 0.38	4.97 ± 0.38**	6.47 ± 0.52	5.77 ± 0.17**	0.13 ± 0.06	0.12 ± 0.12	0.02 ± 0.01	0.02 ± 0.01**	0.87 ± 0.01	0.85 ± 0.02

* and ** denote significant differences at the 0.05 and 0.01 levels, respectively.

Rice grains from CSSLs with low ASV dried similarly to R498, exhibiting better grain integrity. After rehydration, the rice grains could regain their original color and integrity (**Figures 4A, B**). Interestingly, instant rice made from CSSLs with low ASV showed a slight increase in appearance and taste scores, a decrease in hardness, an increase in viscosity, an overall improvement in balance, and a trend of increased comprehensive taste score (**Figure 4B**; **Table 2**). This may be attributed to the resistance to cooking exhibited by rice with low ASV. CSSL-*ALK* ^GGC^-1 had lower appearance and taste scores and higher hardness compared to CSSL-*ALK* ^GGC^-2 (**Table 2**). This finding suggests that the slight difference in taste scores may be attributed to the involvement of SSIIIa in long-chain synthesis, and the increase in B3 chains led to a significant improvement in the overall score of CSSL-*ALK* ^GGC^-2 rice.

In conclusion, we recommend the use of rice with medium to low amylose content, low chalkiness, and low ASV in the production of instant rice.

## Discussion

ECQs are important components of rice quality and are decisive factors in determining the economic value of rice. Previous studies have found that the taste quality of rice is mainly influenced by the amylose content. As the content of amylose increases, the taste quality decreases continuously (Deng et al., 2023; Tian et al., 2009; Zhang et al., 2022). Therefore, in existing standards to evaluate high-quality rice, amylose content is an important indicator to judge taste quality (Tian et al., 2009). Some research suggests that protein content is the second factor affecting taste quality after amylose (Sun et al., 2011). However, other studies have found that an appropriate increase in protein content can improve the palatability of rice (Khan et al., 2020). Additionally, the ASV of rice is also an important indicator of high-quality rice, related to the cooking quality of rice (Zheng et al., 2020). However, whether the ASV affects the appearance, glossiness, and palatability of rice still lacks systematic research. Moreover, chalkiness is an important evaluation index for the appearance quality of rice (Li et al., 2023). Some studies have found a negative correlation between chalkiness and head rice rate with taste quality (Cheng et al., 2005), but direct evidence is still lacking. In this study, we focused on CSSLs with significant variations in amylose content, chalkiness degree, and ASV under the background of R498 (**Figure 1**; **Table 1**; **Supplementary Figure S1**). We also manipulated grain protein contents in R498 seeds through varying levels of nitrogen fertilizer application. Our investigation explored the impact of these four physicochemical characteristics on ECQs under a similar genetic background. The results revealed that starch and protein contents are the main factors affecting the palatability of rice (**Table 1**; **Supplementary Table S4**). Chalkiness, particularly with regard to the WCR, has a significant effect on the integrity of cooked rice while displaying a weak negative correlation with ECQ (**Figure 3A**; **Supplementary Table S4**). ASV influences rice gelatinization temperature and is directly related to the cooking quality of rice but has a minor impact on taste and flavor quality (**Figures 3A, D**; **Table 1**). Based on these findings, we propose standards to enhance the taste and flavor quality of indica rice: relatively low amylose content, low protein content, and low or no chalkiness. These standards have been validated through the assessment of physicochemical properties of 11 newly developed hybrid rice varieties, signifying their importance in the breeding and improvement process of indica rice (**Supplementary Table S5**).

With the improvement of living standards and the acceleration of the pace of life, the demand for instant foods is increasing, mainly centered around instant noodles. However, over 60% of the population in China consume rice as their staple food; hence, in recent years, instant rice has become more popular in the market. Currently, dried instant rice dominates the market, known for its portability, long shelf-life, and ease of consumption. After simple cooking, the flavor, texture, and appearance of instant rice need to be consistent with regular rice. Since it involves secondary cooking, the raw materials used to make instant rice need to be resistant to cooking, and the rice grains after secondary cooking should be intact with distinct contours. However, the quality of products circulating in the current Chinese market is controlled by producers based on their own corporate standards, without national standards. At the same time, there is a lack of systematic research on the factors affecting the resistance to cooking of rice and the integrity of rice grains after secondary cooking. In this study, through the utilization of the identified CSSLs, we have explored the limiting factors in the production process of instant rice (**Figure 4**; **Table 2**). We found that, similar to freshly cooked rice, the taste of instant rice is mainly influenced by amylose content. Rice with medium to low amylose content still maintains a good taste quality after secondary cooking (**Tables 1**, **2**). Additionally, we further discovered that instant rice derived from CSSLs with low ASV exhibits improved color and palatability after rehydration due to their resistance to cooking (**Table 2**). More importantly, our research has unveiled that chalkiness, especially with reference to WCR, significantly impacts the integrity of hydrated rice grains (**Figure 4**). These findings provide valuable theoretical guidance for the selection and breeding of new rice varieties suitable for instant rice processing.

## Data Availability

The original contributions presented in the study are included in the article/**Supplementary Material**. Further inquiries can be directed to the corresponding authors.
