# Supplementary figures and images for: Exploring the impact of key physicochemical properties of rice on taste quality and instant rice processing
Source: Front Plant Sci. 2024 Nov 7;15:1481207. doi: 10.3389/fpls.2024.1481207 (PMC11578832; doi:10.3389/fpls.2024.1481207)

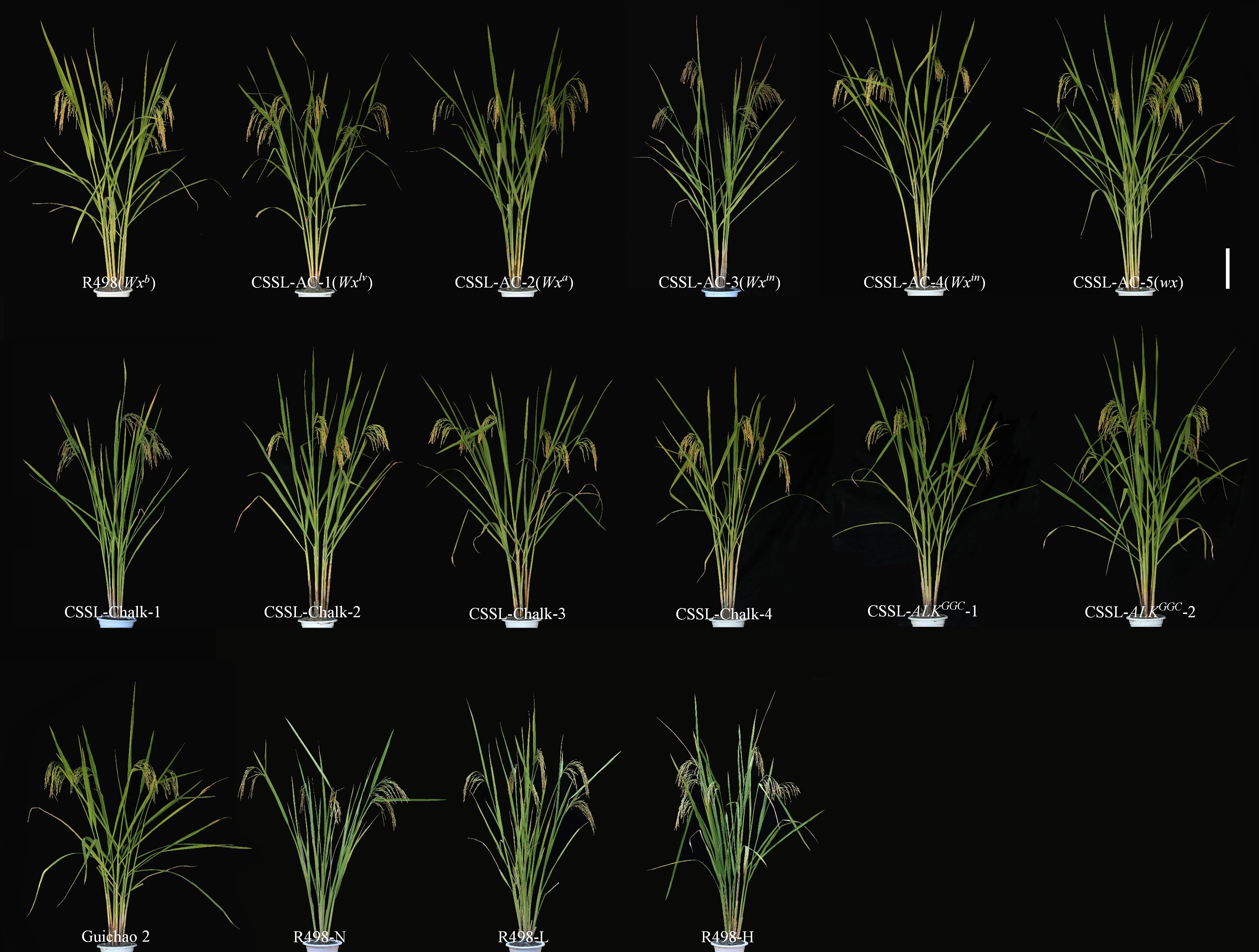

Supplement: Supplementary file 1 [file Image1.jpg]
