# Supplementary material for: Exploring the impact of key physicochemical properties of rice on taste quality and instant rice processing
Source: Front Plant Sci. 2024 Nov 7;15:1481207. doi: 10.3389/fpls.2024.1481207 (PMC11578832; doi:10.3389/fpls.2024.1481207)
Supplement: Supplementary file 2 [file Table1.docx]

**Supplementary Table 1.** Major agronomic traits of lines with R498 and substitutions in each segment

| **Materials** | **PH（cm）** | **PL（cm）** | **TN** | **SSR（%）** | **KGW（g）** | **GL（mm）** | **GW（mm）** |
| --- | --- | --- | --- | --- | --- | --- | --- |
| R498(*Wx^b^*) | 124.70±3.05 | 28.93±1.03 | 7±1 | 89.14±2.59 | 36.90±0.53 | 10.67±0.07 | 2.99±0.03 |
| CSSL-AC-1(*Wx^lv^*) | 122.20±1.75 | 29.41±1.43 | 6±1 | 86.14±1.56* | 36.00±0.52** | 10.42±0.05** | 2.99±0.04 |
| CSSL-AC-2(*Wx^a^*) | 127.60±2.38 | 29.17±1.04 | 7±2 | 86.37±2.17* | 37.11±0.45 | 10.41±0.07** | 2.99±0.03 |
| CSSL-AC-3(*Wx^in^*) | 135.20±2.46** | 23.99±0.85** | 6±1 | 90.14±1.45 | 34.50±0.62** | 10.01±0.08** | 3.06±0.03** |
| CSSL-AC-4(*Wx^in^*) | 138.10±5.64** | 25.26±0.73** | 5±1* | 88.15±2.17 | 33.71±0.58** | 9.91±0.11** | 3.03±0.02** |
| CSSL-AC-5(*wx*) | 133.10±3.85** | 28.14±0.75 | 7±2 | 90.66±2.96 | 33.73±0.45** | 10.36±0.06** | 3.04±0.04** |
| CSSL-Chalk-1（WCR） | 123.70±2.28 | 29.17±0.91 | 6±1 | 83.88±2.16** | 32.87±0.91** | 10.31±0.08** | 2.91±0.04** |
| CSSL-Chalk-2（WBR） | 124.70±2.73 | 26.64±1.01** | 6±2 | 86.15±2.57* | 38.81±0.65** | 10.25±0.11** | 3.36±0.04** |
| CSSL-Chalk-3（WBR） | 126.90±0.89 | 27.46±0.57** | 5±1* | 85.92±2.28** | 37.94±0.63** | 10.09±0.07** | 3.35±0.02** |
| CSSL-Chalk-4（WBR） | 125.50±2.18 | 29.27±0.70 | 5±1* | 86.84±4.06* | 38.09±0.59** | 10.17±0.12** | 3.28±0.05** |
| CSSL-*ALK^G-GC^* -1 | 128.60±2.63 | 27.89±1.04 | 5±2* | 88.83±1.75* | 35.92±0.36** | 10.62±0.09 | 3.06±0.02** |
| CSSL-*ALK^G-GC^* -2 | 132.40±1.56** | 27.30±1.14** | 6±1 | 83.34±2.75** | 34.10±0.49** | 10.33±0.07* | 2.98±0.05 |
| Guichao 2 | 114.80±3.09** | 25.36±0.80** | 9±2** | 87.00±6.34* | 31.54±0.90** | 7.90±0.12** | 3.59±0.05** |
| R498-N | 120.10±3.98 | 26.28±0.77 | 7±0 | 86.34±6.20 | 32.81±1.06 | 10.28±0.20 | 2.97±0.05 |
| R498-L | 116.30±1.78 | 26.46±1.91 | 6±1 | 83.09±6.24** | 33.10±0.99 | 10.32±0.17 | 2.97±0.05 |
| R498-H | 126.90±1.59** | 26.21±0.76 | 9±1** | 84.94±4.61 | 33.35±0.78 | 10.32±0.15 | 2.97±0.05 |

Note: * and ** denote significant differences at the 0.05 and 0.01 levels, respectively.

**Supplementary Table 2.** Sensory evaluation and taste meter test results of 5 distinctive hybrid rice varieties

| **Materials** | **Sensory**  **evaluation** | **Comprehensive** | **Appearance** | **Taste** | **Hardness（kgf）** | **Viscosity（kgf）** | **Balance** | **Elastic** |
| --- | --- | --- | --- | --- | --- | --- | --- | --- |
| **YX2115** | Excellent | 92.00±0.00 | 9.33±0.06 | 9.13±0.06 | 1.85±0.02 | 0.31±0.06 | 0.17±0.03 | 0.91±0.02 |
| **HY584** | Better | 89.00±1.00 | 8.80±0.10 | 8.20±0.20 | 2.78±0.10 | 0.35±0.11 | 0.12±0.03 | 0.90±0.01 |
| **QXY612** | Medium | 86.33±0.58 | 8.37±0.21 | 7.73±0.23 | 2.25±0.18 | 0.29±0.07 | 0.13±0.03 | 0.88±0.01 |
| **FY498** | Poor | 83.33±2.89 | 8.10±0.44 | 7.33±0.38 | 4.47±0.85 | 0.10±0.03 | 0.03±0.01 | 0.88±0.01 |
| **GC2** | Worse | 67.00±1.00 | 6.27±0.12 | 5.67±0.12 | 6.26±0.32 | 0.04±0.02 | 0.01±0.01 | 0.87±0.02 |

**Supplementary Table 3.** The correlation analysis of the measured ECQ indicators

| **Index** | **Comprehensive** | **Appearance** | **Taste** | **Hardness（kgf）** | **Viscosity（kgf）** | **Balance** | **Elastic** |
| --- | --- | --- | --- | --- | --- | --- | --- |
| Comprehensive | 1 |  |  |  |  |  |  |
| Appearance | 0.9857** | 1 |  |  |  |  |  |
| Taste | 0.9734** | 0.9639** | 1 |  |  |  |  |
| Hardness（kgf） | -0.9275** | -0.9025** | -0.9690** | 1 |  |  |  |
| Viscosity（kgf） | 0.7942** | 0.8376** | 0.7853** | -0.6706** | 1 |  |  |
| Balance | 0.7493** | 0.7814** | 0.7320** | -0.7018** | 0.8752** | 1 |  |
| Elastic | 0.6579** | 0.5729** | 0.7404** | -0.8488* | 0.2610** | 0.3089** | 1 |

* and ** denote significant differences at the 0.05 and 0.01 levels, respectively. r | < 0.3, the two indexes are not related; 0.3 < | r | < 0.5, the two indexes were weakly correlated; 0.5 < | r | < 0.8, the two indexes were moderately correlated, and | r | > 0.8, the two indexes were strongly correlated.

**Supplementary Table 4.** The correction analysis of physicochemical characters and ECQs of each isolated CSSLs

| **Index** | **CGP (%)** | **CGG（%）** | **PC（%）** | **FC（%）** | **AC（%）** | **Comprehensive** | **Appearance** | **Taste** | **Hardness（kgf）** | **Viscosity（kgf）** | **Balance** | **Elastic** |
| --- | --- | --- | --- | --- | --- | --- | --- | --- | --- | --- | --- | --- |
| **CGP (%)** | 1 |  |  |  |  |  |  |  |  |  |  |  |
| **CGG（%）** | 0.7929** | 1 |  |  |  |  |  |  |  |  |  |  |
| **PC（%）** | 0.7096** | 0.5027** | 1 |  |  |  |  |  |  |  |  |  |
| **FC（%）** | 0.3702** | 0.2181** | 0.2902** | 1 |  |  |  |  |  |  |  |  |
| **AC（%）** | 0.1512** | -0.1676** | 0.2146** | -0.4743** | 1 |  |  |  |  |  |  |  |
| **Comprehensive** | -0.3235** | -0.3369** | -0.6069** | 0.2775** | -0.6356** | 1 |  |  |  |  |  |  |
| **Appearance** | -0.4883** | -0.3611** | -0.5890** | 0.3194** | -0.5772** | 0.9763** | 1 |  |  |  |  |  |
| **Taste** | -0.5498** | -0.3501** | -0.6325** | 0.2587** | -0.6653** | 0.9733** | 0.9743 | 1 |  |  |  |  |
| **Hardness（kgf）** | 0.3020** | 0.1348** | 0.4617** | -0.4243** | 0.6771** | -0.9398** | -0.9292** | -0.8946** | 1 |  |  |  |
| **Viscosity（kgf）** | -0.5561** | -0.4396** | -0.4133** | 0.3441* | -0.6936** | 0.7424** | 0.7234** | 0.7985** | -0.5931** | 1 |  |  |
| **Balance** | -0.3638** | -0.1471** | -0.3115** | 0.5300** | -0.8965** | 0.7376** | 0.7050** | 0.7841** | -0.6978** | 0.9031** | 1 |  |
| **Elastic** | 0.0125** | -0.0192** | -0.1921** | 0.1155** | 0.0744** | 0.3604** | 0.3433** | 0.2204** | -0.5027** | -0.2328** | -0.1314** | 1 |

Note: * and ** denote significant differences at the 0.05 and 0.01 levels, respectively. | r | < 0.3, the two indexes are not related; 0.3 < | r | < 0.5, the two indexes were weakly correlated; 0.5 < | r | < 0.8, the two indexes were moderately correlated, and | r | > 0.8, the two indexes were strongly correlated

**Supplementary Table 5.** Determination of physicochemical qualities and ECQ of ten hybrid rice combinations

| **Hybrid rice variety** | **ASV** | **CGP(%)** | **PC（%）** | **FC（%）** | **AC（%）** | **Comprehensive** | **Appearance** | **Taste** | **Hardness（kgf）** | **Viscosity（kgf）** | **Balance** | **Elastic** |
| --- | --- | --- | --- | --- | --- | --- | --- | --- | --- | --- | --- | --- |
| Quanxiang1A /Shuhui16 | 7 | 0.5 | 6.47±0.05 | 0.81±0.08 | 16.3 | 79.33±1.15 | 7.73±0.12 | 6.90±0.10 | 2.48±0.21 | 0.38±0.07 | 0.15±0.03 | 0.89±0.02 |
| Quanxiang3A /Luhui612 | 7 | 0.9 | 5.92±0.04 | 0.45±0.09 | 15.2 | 80.67±0.58 | 7.73±0.06 | 6.93±0.06 | 1.94±0.14 | 0.29±0.10 | 0.15±0.05 | 0.89±0.01 |
| S28-13 / Yuhe | 4.3 | 1.1 | 5.94±0.09 | 0.42±0.02 | 16 | 84.67±0.58 | 8.43±0.06 | 7.37±0.06 | 1.69±0.10 | 0.35±0.08 | 0.21±0.04 | 0.90±0.00 |
| Quanxiang3A / Shuhui882 | 6.7 | 3.1 | 7.15±0.01 | 1.06±0.15 | 15.6 | 77.33±0.58 | 7.43±0.15 | 6.47±0.21 | 2.38±0.22 | 0.40±0.90 | 0.17±0.02 | 0.86±0.00 |
| Quanxiang1A /Shuhui882 | 6.7 | 3.1 | 7.43±0.15 | 0.91±0.00 | 15.6 | 72.33±0.58 | 6.70±0.10 | 5.90±0.10 | 2.55±0.09 | 0.36±0.05 | 0.14±0.01 | 0.88±0.01 |
| L66 / Shuhui308 | 7 | 2.4 | 5.27±0.14 | 0.95±0.08 | 16 | 79.00±1.73 | 7.10±0.20 | 6.47±0.15 | 2.17±0.25 | 0.26±0.03 | 0.11±0.02 | 0.91±0.00 |
| Jincheng3A  / Shuhui91 | 7 | 0.4 | 5.74±0.30 | 0.76±0.12 | 19.1 | 77.67±0.58 | 7.43±0.12 | 6.57±0.15 | 2.30±0.13 | 0.20±0.03 | 0.09±0.01 | 0.91±0.02 |
| Jincheng2A / Shuhui91 | 7 | 1 | 5.52±0.03 | 0.69±0.00 | 19.1 | 78.00±1.00 | 7.43±0.21 | 6.57±0.15 | 2.43±0.17 | 0.22±0.01 | 0.09±0.01 | 0.89±0.02 |
| HS59S / Shuhui586 | 4.6 | 3.5 | 6.17±0.12 | 0.46±0.07 | 15.3 | 79.67±0.58 | 7.63±0.15 | 6.67±0.06 | 1.62±0.12 | 0.34±0.07 | 0.21±0.03 | 0.92±0.01 |
| Quanxiang3A / Shuhui569 | 6.7 | 1.7 | 7.23±0.19 | 0.73±0.03 | 15.5 | 71.67±0.58 | 6.83±0.06 | 5.93±0.15 | 2.41±0.12 | 0.36±0.08 | 0.15±0.03 | 0.88±0.02 |

**Supplementary Table 6.** Primers for the identification of *Wx* and *ALK*

| Primer Name | Primer suquence(5'-3') | Gene ID | Purpose |
| --- | --- | --- | --- |
| SY575 | TTTGGCTCTGAGGCACT | LOC_Os06g04200 | Detect the difference in the first base of the first intron of *Wx*. |
| SY568 | TTTGACCAACTCGGCTA | LOC_Os06g04200 | Detect the difference in the first base of the first intron of *Wx*. |
| AK823 | TGCAGAGATCTTCCACAGCA | LOC_Os06g04200 | Detect the 23bp difference in the second exon of *Wx*. |
| AK824 | GCTGGTCGTCACGCTGAG | LOC_Os06g04200 | Detect the 23bp difference in the second exon of *Wx*. |
| SY547 | GTTGTGGCTGAGGTAGGA | LOC_Os06g04200 | Detect the base differences in exons 4, 5, and 6 of *Wx*. |
| SY548 | CATTGGGCTGGTAGTTGT | LOC_Os06g04200 | Detect the base differences in exons 4, 5, and 6 of *Wx*. |
| SY549 | GGTGGAGGGCAGGAAGAT | LOC_Os06g04200 | Detect the base difference in exon 10 of *Wx*. |
| SY550 | AGCACAGGGCTGGAGAAA | LOC_Os06g04200 | Detect the base difference in exon 10 of *Wx*. |
| ZT140F | GAGCCGTGCGGCCTCAACCA | LOC_Os06g12450 | Discriminate the functional SNPs of TT at 4329/4330 bp, BamHⅠ cut GC allele |
| ZT140R | CCTGCGACATGCCGCGCACCTGGAT | LOC_Os06g12450 | Discriminate the functional SNPs of TT at 4329/4330 bp, BamHⅠ cut GC allele |
| ZT132F | GAGCCGTGCGGCCTCAACCA | LOC_Os06g12450 | Detect the difference in the eighth exon of *ALK*. |
| ZT132R | ACCGCGGGCGGACATGGTCT | LOC_Os06g12450 | Detect the difference in the eighth exon of *ALK*. |
